# SeqSNP-Based Targeted GBS Provides Insight into the Genetic Relationships among Global Collections of *Brassica rapa* ssp. *oleifera* (Turnip Rape)

**DOI:** 10.3390/genes15091187

**Published:** 2024-09-10

**Authors:** Mulatu Geleta, Jagadeesh Sundaramoorthy, Anders S. Carlsson

**Affiliations:** Department of Plant Breeding, Swedish University of Agricultural Sciences, P.O. Box 190, 23422 Lomma, Sweden; jagadeesh@slu.se (J.S.); anders.carlsson@slu.se (A.S.C.)

**Keywords:** genetic differentiation, genic SNPs, oilseed crop, private alleles, targeted-GBS, turnip rape

## Abstract

Turnip rape is a multi-purpose crop cultivated in temperate regions. Due to its ability to fit into crop rotation systems and its role as a food and feed source, spring-type turnip rape cultivation is on the rise. To improve the crop’s productivity and nutritional value, it is essential to understand its genetic diversity. In this study, 188 spring-type accessions were genotyped using SeqSNP, a targeted genotyping-by-sequencing method to determine genetic relationships between various groups and assess the potential effects of mutations within genes regulating major desirable traits. Single nucleotide polymorphism (SNP) alleles at six loci were predicted to have high effects on their corresponding genes’ functions, whereas nine loci had country/region-specific alleles. A neighbor-joining cluster analysis revealed three major clusters (I to III). About 72% of cluster-I accessions were of Asian origin, whereas 88.5% of European accessions and all North American accessions were placed in cluster-II or cluster-III. A principal coordinate analysis explained 65.3% of the total genetic variation. An analysis of molecular variance revealed significant differentiation among different groups of accessions. Compared to Asian cultivars, European and North American cultivars share more genetic similarities. Hence, crossbreeding Asian and European cultivars may result in improved cultivars due to desirable allele recombination. Compared to landraces and wild populations, the cultivars had more genetic variation, indicating that breeding had not caused genetic erosion. There were no significant differences between Swedish turnip rape cultivars and the NordGen collection. Hence, crossbreeding with genetically distinct cultivars could enhance the gene pool’s genetic diversity and facilitate superior cultivar development.

## 1. Introduction

The genus *Brassica* L., which belongs to the tribe Brassiceae, is one of the most economically significant genera in the family Brassicaceae. Genetic relationships between cultivated *Brassica* species were outlined by the classical triangle of U, which includes three diploid and three allotetraploid species [1]. *B. rapa*, *B. nigra*, and *B. oleracea* are the three diploid species, with diploid (2 n) chromosome numbers 20, 16, and 18, and genome designations of AA, BB, and CC, respectively. Allopolyploidization led to the evolution of three tetraploid species: *B. napus* (AACC, 2 n = 38), *B. juncea* (AABB, 2 n = 36), and *B. carinata* (BBCC, 2 n = 34).

*B. rapa* L. (AA, 2 n = 20) is probably the earliest domesticated among the members of the triangle of U [2]. It has been cultivated as an oil and vegetable crop worldwide for its high nutritional and economic significance. During its domestication and cultivation history, it has been diversified into different morphotypes (leafy vegetables, swollen root vegetables, and oilseed crops), which were further classified into different subspecies [3,4]. According to McAlvay et al. [5], swollen root vegetables (turnips) were likely the first to be domesticated among the different morphotypes, from which leafy types were developed from distinct lineages in East Asia and Europe.

Oilseed morphotypes include *B. rapa* ssp. *oleifera* (turnip rape), *B. rapa* ssp. *dichotoma* (brown sarson), *B. rapa* ssp. *trilocularis* (yellow sarson), and *B. rapa* ssp. *rapa* (turnip) [2,6], of which turnip rape is the major one. Despite that molecular data strongly suggest that turnip rape originated from multiple lineages, the specific progenitors of the different lineages remain unclear [4,7]. Probably domesticated directly from their wild progenitors as root vegetables, turnips are very old in Europe [5,8]. According to Reiner et al. [8], European turnip rape is closely related to turnips genetically and may have evolved from them through selection. As increased oil production became necessary in some temperate regions in Europe starting around the 14th century, oilseed varieties of the turnip rape (*B. rapa* L. ssp. *oleifera* (DC.) Metzg.) were chosen [8]. Both winter and spring varieties have been developed and cultivated in Europe [9,10,11]. 

Forage and fodder crops are predominant in crop rotation in Northern European agriculture. It is well understood that for plant cultivation to be sustainable, good crop rotation is required, which contributes to pest and disease control while also promoting biodiversity. It is not sufficient with one or two crops in crop rotation to achieve sustainable production. Including oil crops in crop rotation systems is one way to improve crop rotation. Spring turnip rape has the potential to be a well-adapted and environmentally sustainable oil crop in crop rotations [10,12]. Spring turnip rape is also known to growers as an important interruption crop in the northern crop rotation, and the use of turnip rape oil and cake in the food and feed industries, respectively, is well known [13]. 

One of the advantages of the spring turnip rape cultivars is that they mature earlier than early-maturing oilseed rape cultivars [13,14]. As a result, it is the preferred oil crop in temperate countries in the northern hemisphere. Spring turnip rape has been an important oilseed crop in several countries at various times. It has been the most important oilseed crop in Finland for many years [10]. In Sweden, turnip rape was bred for a long time and even became the largest oil crop in terms of production area for a few years in the 1980s, being grown in the temperate latitudes of the northern hemisphere ranging from 55° N to 65° N. However, its breeding program was discontinued in 2002 [15], leading to a lack of improved spring turnip rape cultivars available to farmers. This resulted in a decrease in turnip rape production area to about 15,000 hectares, according to the Swedish Board of Agriculture report [16]. Hence, due to drastically reduced plant breeding efforts of turnip rape during the last 30 years, it has fallen behind other crops in productivity. Plant breeding, as is widely known, contributes to the development of improved crops with high yields and other desirable traits, and combining many of these crops in rotation systems contributes to healthier crops with overall higher yields. Including improved spring turnip rape in crop rotation is an important strategy to strengthen agricultural production in various parts of the world, including Northern Europe.

Several gene banks throughout the world have extensive and well-evaluated collections of Brassica species. Diverse spring turnip rape collections exist in various gene banks. These collections possess useful genetic variation that can be tapped into the crop’s breeding programs. A rigorous genetic characterization, assessment of genetic diversity, and identification of potential putative genes that could be exploited in the breeding programs are essential for optimal utilization of these collections. In line with this, various types of molecular markers have been utilized for the characterization of crop genetic resources and the determination of their genetic diversity [17,18,19,20,21,22,23]. 

In *B. rapa*, a variety of molecular marker types, including restriction fragment length polymorphism (RFLP), random amplified polymorphic DNA (RAPD), amplified fragment length polymorphism (AFLP), and simple sequence repeat (SSR) have been used in population genetic and phylogenetic studies to clarify the relationships within and among its subspecies [24,25,26,27,28,29,30,31,32]. Recent research has utilized whole-genome sequencing and single nucleotide polymorphism (SNP) genotyping methods for genetic analyses in this species [33,34]. Such research is crucial for the efficient conservation of crop genetic resources and plant breeding since genetic variation is a crucial source of novel valuable alleles for crop improvement by breeders [35,36]. Therefore, population genetic analyses of the collections of spring turnip rape genetic resources for more efficient conservation and utilization in plant breeding is highly desirable. In this study, an SNP-based evaluation of a well-balanced collection of spring turnip rape was conducted to determine its genetic diversity and germplasm exchange status. In addition, we evaluated the effects of genic SNPs on their genes.

## 2. Materials and Methods

### 2.1. Plant Materials and Growth Conditions

A total of 188 accessions obtained from different sources (gene banks and seed and breeding companies) were investigated in this study (Table 1 and Table S1). These include thirty-four accessions from NordGen (the Nordic gene bank), thirty accessions from PGRC (Plant Genetic Resources Centre, Canada), eleven accessions from CGN (Centre for Genetic Resources, The Netherlands), eighty-six accessions from IPK (Genebank—Leibniz-Institut, Germany), twelve accessions from NPGS (The National Plant Germplasm System, USA), thirteen accessions from Jerrestad Agro AB (a turnip rape breeding company, Sweden), and two accessions from A&A Canada (A&A Seed Farms, Canada). The accessions comprised wild populations, landraces, breeding populations, and cultivars. The focus was on collecting turnip rape accessions of spring type. The breeding status (population type) of 34 accessions was described as “unknown”, while 12 accessions were described as either breeders’ variety or breeding/research material or improved material (Table S1). The other accessions are cultivars (ninety-four), landraces (forty-one), and wild populations (seven). 

Seeds of each accession were planted in a greenhouse at the Swedish University of Agricultural Sciences (SLU, Alnarp, Sweden; 55°65′ N, 13°07′ E) for DNA extraction. For this purpose, two 2 L plastic pots filled with soil were used. Leaf tissue sampling from young seedlings for DNA extraction was conducted three weeks after planting. Each accession was represented by 20 plants for optimal representation of their genetic variation. For sampling the leaf tissue, a BioArk Leaf collection kit provided by LGC, Biosearch Technologies (https://biosearchassets.blob.core.windows.net/assetsv6/guide_bioark-leaf-collection-kit.pdf (accessed on 19 August 2024)) was used. From each plant, a single 6 mm leaf disk was sampled using a punch, and leaf disks from ten plants of each accession were pooled into a sampling plate. Hence, each accession was represented by two leaf-tissue pools, each of which was sampled from ten plants. The collected leaf tissue was then sent to LGC, Biosearch Technologies (Berlin, Germany) for DNA extraction and subsequent genotyping. Using the Sbeadex plant kit (https://biosearch-cdn.azureedge.net/assetsv6/sbeadex-plant-data-sheet.pdf (accessed on 19 August 2024)), high-quality genomic DNA was extracted for SeqSNP genotyping.

### 2.2. SNP Loci Selection, SeqSNP Assay Design and Target Sequencing

Turnip rape SNP markers were selected from the CropSNPdb database (http://snpdb.appliedbioinformatics.com.au/ (accessed on 19 August 2024); [37]). Genes reported to regulate agronomic and nutritional content traits and those regulating responses to biotic and abiotic stresses were prioritized for SNP selection. The distribution of SNPs across the ten *B. rapa* chromosomes was an additional criterion for SNP selection. SNPs within genes of interest were prioritized, although SNPs near genes of interest were also included when mutations were absent within the genes of interest. For the SeqSNP assay design, 1280 bi-allelic SNPs were selected, including SNPs within genes regulating disease resistance and oil content. *B. rapa* genome CAAS_Brap_v3.01 (https://www.ncbi.nlm.nih.gov/assembly/GCF_000309985.2 (accessed on 19 August 2024)) was used as a reference. Among the 1280 target SNPs, 1009 were fully covered (two oligo probes per target) and passed the high-specificity assay design, 245 were partially covered (one oligo probe per target), and 26 failed the design. A total of 400 bi-allelic SNPs were selected from the 1009 SNPs that passed the high-specificity assay design for SeqSNP analysis, taking into account the distribution of the SNPs across the genome and the function of the genes. These include 29 and 35 SNPs within and nearby, respectively, genes known to regulate disease resistance or oil content. The other 336 SNPs are located within other *B. rapa* genes. SeqSNP kit containing 800 high-specificity oligo probes for 400 SNPs was then produced, a sequencing library was constructed, and the target SNPs were sequenced. Illumina NextSeq 500/550 v2 platform was used to sequence 150 base pairs (bp) in single read mode. An average of 215,696 reads were generated per sample, and an average of 473× coverage was achieved for target SNPs.

### 2.3. De Novo SNP Discovery, Target and Novel SNP Allele Counting, and Data Filtering 

Following sequencing of the target regions, the raw reads were adapter-clipped and quality-trimmed where reads containing Ns were removed, reads were trimmed to obtain a minimum average Phred quality score of 30 over a window of ten bp and reads with final length < 130 bp were discarded. 

Bowtie2 v2.2.3 [38] was used to align quality-trimmed reads against the reference genome for variant detection and allele counts. Alleles with an allele count of less than eight were set to zero, according to the threshold set for the variant discovery pipeline, to exclude alleles recorded due to a potential sequencing error. 

Out of the 400 target SNP loci, 25 had only one allele across the 188 accessions, and hence, the accessions were monomorphic at these loci. Two alleles matching the original SNPs were recorded at 362 loci, while three alleles were recorded at 13 loci (one additional allele was discovered at each of these loci). In addition to the target SNPs, 211 SNPs were discovered de novo near the target SNPs (within 75 bp regions on either side of the target SNPs), of which 201 and 10 were bi-allelic and tri-allelic, respectively. For simplicity, only bi-allelic SNPs were used for further data analysis.

In total, 563 bi-allelic SNPs were obtained, of which 29 bi-allelic SNPs with over 5% missing data were excluded from further data analysis. Hence, 534 bi-allelic SNPs (341 target SNPs and 193 de novo discovered SNPs) with less than 5% missing data were used for further analysis. The minimum allele frequency (MAF) of this set of markers ranged from 0.003 to 0.499.

### 2.4. Converting Allele Counts into Allele Frequencies

The allele counts of bi-allelic loci were converted to allele frequency data. Since each accession was represented by two samples (each a pool of ten genotypes), an analysis of variance was carried out to determine if there were significant differences between the two sample sets, which revealed no significant differences. Therefore, even when genotype pools were sequenced, the SeqSNP method proved to be reliable. The average allele frequencies of the accessions at the SNP loci were used for further data analysis. Interestingly, 91 of the 188 accessions had only one allele at 22 of the 534 loci. As a result, all genotypes within each of these 91 accessions were homozygous for the same allele at each of those 22 loci. These 22 loci were analyzed separately using genotypic data (instead of allele frequency data) and the results were compared with those obtained using allele frequency data.

### 2.5. Cluster and Principal Coordinate Analyses, Analysis of Molecular Variance (AMOVA), and Variant Analysis

Different statistical analyses were carried out based on allelic frequency data of 534 SNP loci for the 188 accessions. Nei’s unbiased genetic distance [39] between the 188 accessions was calculated using the NTSYSpc program [40]. Nei’s unbiased genetic distance was used to perform neighbor-joining cluster analysis [41] using MEGA7 [42], whereas GenAlEx version 6.5 software [43] was employed for Nei’s unbiased genetic distance-based principal coordinate analysis (PCoA). The number of country-specific and region-specific alleles (private alleles) was calculated using GenAlEx version 6.5. The percent polymorphic loci for different population types (cultivars, landraces, and wild populations) was also determined using GenAlEx.

Analysis of molecular variance (AMOVA) was conducted using Arlequin version 3.5 [44] based on allele frequency data of 534 loci by grouping the accessions into different groups according to their germplasm provider, population type, country of origin, or region of origin. AMOVA was also conducted based on genotypic data of 22 SNP loci that lack heterozygosity for different numbers of turnip rape accessions grouped according to their germplasm provider, population type, country of origin, or region of origin. In addition, allele frequency data and genotypic data were used to perform pairwise genetic differentiation between the accessions grouped according to the four aforementioned criteria using Arlequin version 3.5.

The SNP variants were analyzed and their functional consequences were predicted via the Variant Effect Predictor (VEP) tool integrated into EnsemblPlants (https://plants.ensembl.org/tools.html (accessed on 19 August 2024)).

## 3. Results

**Figure 1:**
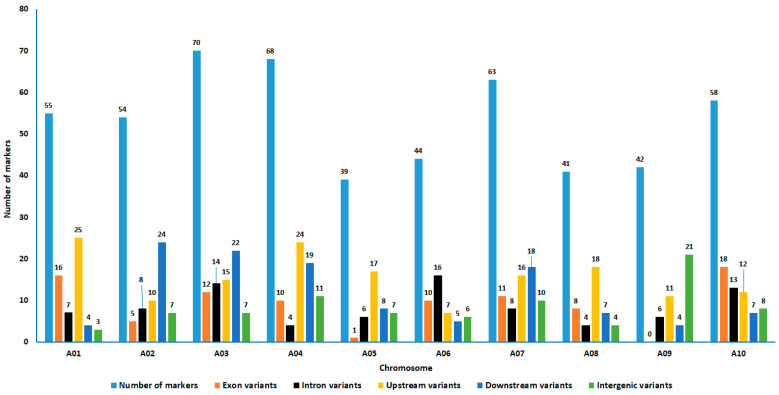
The distribution of the 534 bi-allelic SNPs used in this present study across the ten turnip rape chromosomes, including both genic (intron and exon) and non-genic (upstream, downstream, and intergenic) SNPs. The SNP markers covered 27.5 Mbp, 29.7 Mbp, 31.0 Mbp, 21.7 Mbp, 28.4 Mbp, 26.2 Mbp, 26.7 Mbp, 19.2 Mbp, 41.1 Mbp, and 20.1 Mbp regions on chromosomes A01, A02, A03, A04, A05, A06, A07, A08, A09, and A10, respectively.

The final set of 534 SNP markers used for data analysis, comprising 341 originally targeted SNPs and 193 de novo discovered SNPs, were distributed across the ten *B. rapa* chromosomes. The genomic regions covered on each chromosome ranged from 19.2 Mbp (in chromosome A08) to 41.1 Mbp (in chromosome A09). The SNP markers included 91 exons, 86 introns, 155 upstream genes, 118 downstream genes, and 84 intergenic variants across the ten chromosomes. The number of markers per chromosome ranged from 39 (chromosome A05) to 70 (chromosome A03) (Figure 1). The number of exon variants per chromosome ranged from zero (chromosome A09) to 18 (chromosome A10). 

Of the 534 SNPs, 108 SNPs within coding sequences and those playing roles in gene splicing are provided in Table 2, together with the potential consequences of the SNPs. These include missense variants (sixty-six SNPs), synonymous variants (twenty-one SNPs), stop-retained variants (one SNP), and stop-lost variants (one SNP), as well as nineteen SNPs that play roles in gene splicing (Table 2). According to the variant effect predictor (VEP), the 66 missense variants and the stop-retained variant have moderate effects on the functions of their corresponding genes. Six of the one hundred and eight SNP variants are predicted to have a high effect on their corresponding genes’ functions. These are three splice donor variants (on chromosomes A03, A06, and A07), two splice acceptor variants (on chromosomes A03 and A06), and one stop-lost variant (on chromosome A04) (Table 2).

**Figure 2:**
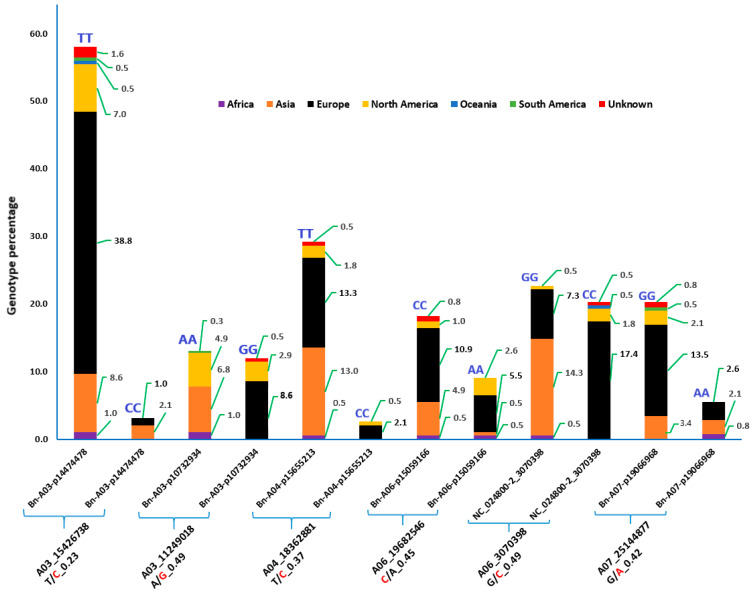
Bar graphs depicting the distribution of homozygous genotypes at six SNP loci with predicted high-effect mutations (stop lost, splice acceptor variant, or splice donor variant) across accessions from different geographical regions. Marker names are given above the blue square brackets, whereas the corresponding chromosomes, SNP positions, and SNP alleles are given below the brackets. Minor alleles are shown in red together with their frequencies. Corresponding genotypes are shown on each bar. Values within the figures are the percentages of homozygous genotypes for each region where the corresponding alleles were recorded.

The minor allele frequencies of the six loci that carry alleles with predicted high effects on their genes ranged from 0.23 to 0.49 (Figure 2). Except for locus Bn-A06-p15059166, where the alternate allele had a frequency of 0.55, the minor alleles are alternate alleles predicted to have a high effect on their genes.

Two of the six loci (Bn-A03-p14474478 and Bn-A03-p10732934) that carry alleles with predicted high effects on their genes are located on chromosome A03. At locus Bn-A03-p14474478, all individuals in 58.1% of the accessions were TT homozygotes, of which European accessions accounted for 38.8%, while those with CC homozygotes accounted for only 3.1%. Accessions with CC genotypes were recorded only in Asian (2.1%) and European accessions (1%) (Figure 2). At locus Bn-A03-p10732934, all individuals in 13% of the accessions were AA homozygotes, while those with GG homozygotes accounted for 12%. GG homozygotes were recorded only in European (8.6%) and Asian accessions (2.9%). The third locus (Bn-A04-p15655213) is located on chromosome A04. At this locus, 29.2% of the accessions contained only TT homozygotes, while 2.6% contained only CC homozygotes. CC genotypes were recorded only in European (2.1%) and North American (0.5%) accessions (Figure 2).

Among the remaining three loci that carry alleles with predicted high effects on their genes, two (Bn-A06-p15059166 and NC_024800-2_3070398) are located on chromosome A06, while one (Bn-A07-p19066968) is located on chromosome A07. At locus Bn-A06-p15059166, all individuals in 18.2% of the accessions were CC homozygotes, while 9.1% had only AA homozygotes. The AA genotypes were recorded in European (5.5%), North American (2.6%), African (0.5%), and Asian (0.5%) accessions (Figure 2). At locus NC_024800-2_3070398, all individuals in 22.7% of the accessions were GG homozygotes, while 20.3% of the accessions had only CC homozygotes. CC homozygotes were detected in accessions from Europe (17.4%), North America (1.8%), and Africa (0.5%). At locus Bn-A07-p19066968, in 20.3% of the accessions, all individuals were homozygous GG, while in 5.5% of the accessions, all individuals were homozygous AA. The AA homozygotes were recorded in European (2.6%), Asian (2.1%), and African (0.8%) accessions (Figure 2).

### 3.1. Cluster Analysis

**Figure 3:**
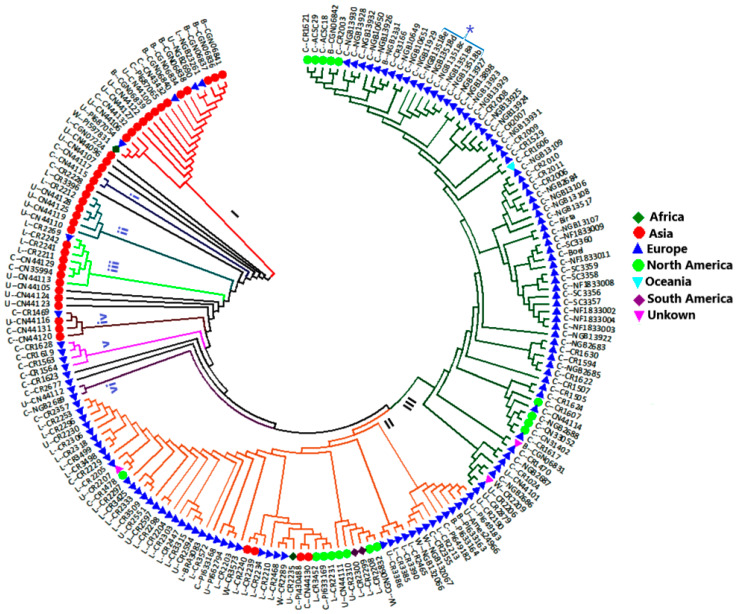
Neighbor-joining tree of 188 turnip rape accessions generated based on Nei’s unbiased genetic distance estimated using 534 SNP loci. The tree comprises three major clusters (I to III), six minor clusters (i to vi), and nine solitary accessions. Accessions from different regions are represented by different labels, as shown in the key to the right of the tree. Note: one accession (NGB13518) shown with a square bracket and an asterisk had five replicates (NGB13518a to NGB13518e) as a quality-control measure of the sequencing methods used.

The Nei’s unbiased genetic distance-based cluster analysis using the neighbor-joining method resulted in an optimal tree of 188 turnip rape accessions comprising three major clusters (I to III), six minor clusters (i to vi), and nine solitary accessions. The three major clusters (I, II, and III) comprised 18, 62, and 74 accessions, respectively. The six minor clusters (i to vi) comprised two, six, seven, four, four, and two accessions, in that order (Figure 3). 

In cluster-I, 72.2% of the accessions were of Asian origin. In the case of minor clusters, all the accessions in clusters-i to iv, except one in cluster-iii, were of Asian origin. Additionally, seven of the nine solitary accessions were of Asian origin, and all of them were closer to the base of the tree than the remaining two accessions. The remaining four accessions of Asian origin were clustered with other accessions in cluster-II.

In cluster-II, 74.2% and 12.9% of the accessions were of European and North American origin, respectively. European and North American accessions accounted for 83.8% and 12.2% of cluster-III accessions, respectively. All accessions in the cluster-v and cluster-vi were of European origin. Overall, 88.5% of European accessions were placed in cluster-II or cluster-III.

All North American accessions were placed in cluster-II or cluster-III. The two South American accessions were clustered closely together with four North American accessions within cluster-II. On the other hand, the two African accessions were placed separately in cluster-I and cluster-II. The single accession (cultivar CR1529) from Oceania (Australia) was closely clustered with a European (German) accession (cultivar 1606) within cluster-III.

Cultivars account for 16.7% of cluster-I, 14.5% of cluster-II, and 90.5% of cluster-III. Thirty-three of the forty-one landrace accessions (80.5%) were clustered in cluster-II, accounting for 53.2% of accessions in this cluster. Four of the seven accessions in cluster-iii were also landrace accessions. Five of the seven wild accessions were clustered in cluster-II. The remaining two were placed in cluster-I and cluster-III, respectively.

All cultivars obtained from NordGen (which account for 85.3% of the accessions obtained from this gene bank) were clustered in cluster-III, with one exception. The most distinct cultivars of European origin were CR1469 (cluster-iv; Germany), the four accessions in cluster-v (Germany), CR1623 (Sweden), and CR2677 (Germany), all obtained from IPK. Interestingly, all 13 cultivars from the Swedish turnip rape breeding company Jerrestad AB were clustered closely together within cluster-III, with the most distinct one being Birta, which is almost genetically identical to accession NGB13107 (a cultivar from NordGen) (Figure 3).

It is worth noting that Asian cultivars were more diverse than those from Europe and North America. NGB23261 from Finland was the most distinct European landrace accession, clustered closely with Asian accessions in cluster-I. Among the wild populations, the most distinct accession was PI597831 from Egypt, which was placed in cluster-I, dominated by Asian accessions.

### 3.2. Principal Coordinate Analysis

**Figure 4:**
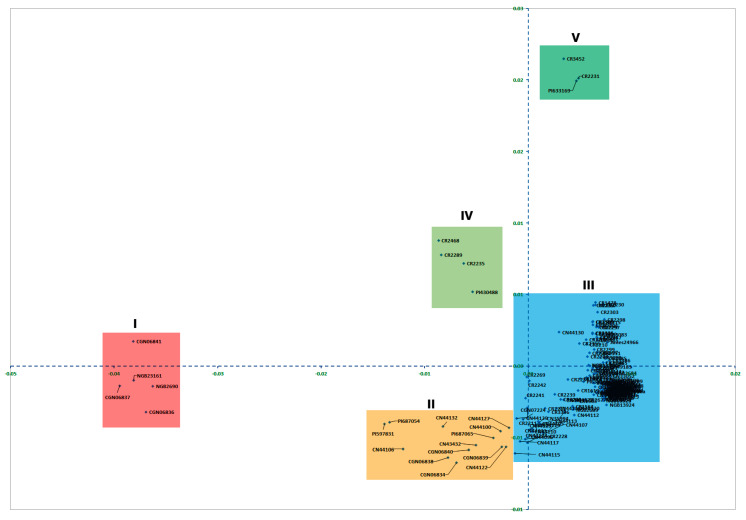
Principal coordinate analysis (PCoA)-based two−dimensional plot for 188 accessions that formed five clusters, in which the first and second axes explained 55.4% and 9.9% of the total variation, respectively.

The first and second principal coordinates (PCo1 and PCo2) explained 55.4% and 9.9% of the total variation among the 188 turnip rape accessions, with the two together accounting for 65.3% of the total variation. Taking into consideration both PCo1 and PCo2, the 188 accessions formed five clusters (I to V) (Figure 4). Cluster-I (five accessions) was the most distinct group clearly separated from all other clusters along PCo1, which accounted for over half of the total variation (55.4%). This cluster was separated from cluster-IV and cluster-V along PCo1 and PCo2. Cluster-II (13 accessions) was separated from cluster-I, cluster-III, and cluster-V along both PCo1 and PCo2 while separated from cluster-IV along PCo2. Similarly, cluster-III (a major cluster containing 163 of the 188 accessions; 86.7%) was separated from cluster-I, cluster-II, and cluster-IV along both PCo1 and PCo2 while separated from cluster-V along PCo2. PCo1 and PCo2 separated cluster-IV (4 accessions) from cluster-I, cluster-III, and cluster-V, while PCo2 separated it from cluster-II. Similarly, cluster-V (3 accessions) was separated from cluster-I, cluster-II, and cluster-IV along both PCo1 and PCo2 while separated from cluster-III along PCo2.

The five accessions in cluster-I of the PCoA biplot were those closely clustered within cluster-I of the neighbor-joining (NJ) tree. Similarly, the 13 accessions in cluster-II of the PCoA biplot are the remaining members of cluster-I of the NJ tree (Figure 4). The PCoA bi-plot cluster-III comprised all accessions that were not members of the NJ tree cluster-I, except seven accessions that are members of cluster-II of the NJ tree. Four of these seven accessions that were closely clustered within cluster-II of the NJ tree formed cluster-IV in the PCoA biplot. The other three accessions formed cluster-V in the PCoA biplot (Figure 4).

### 3.3. Analysis of Molecular Variance (AMOVA)

The analysis of molecular variance (AMOVA) was conducted based on allele frequency data of 534 bi-allelic SNP markers. This includes a fixation index FST, which measures population differentiation, whose maximum value of one is attained when the populations are fixed for different alleles. AMOVA revealed highly significant differentiation (*p* < 0.01) between groups of accessions grouped according to their germplasm provider, population type, country of origin, and region of origin, accounting for 19%, 17.5%, 38%, and 27% of the total genetic variation, respectively (Table 3). Similarly, AMOVA conducted on the genotypic data of 22 bi-allelic loci lacking heterozygosity in 91 of the 188 accessions revealed significant differentiation (*p* < 0.01) between groups of accessions grouped according to their germplasm provider, population type, country of origin, and region of origin, accounting for 23.5%, 17.1%, 51.1%, and 59% of the total genetic variation, respectively (Table 4).

Pair-wise differentiation analysis between different population types based on the allele frequency data of 534 SNP loci showed significant differentiation (*p* < 0.05) between cultivars and landraces as well as between cultivars and wild populations (Table 5). However, there was no significant differentiation between landraces and wild populations. Significant differentiation between cultivars and landraces was also evident when the genotypic data from the 22 loci were analyzed (Table 6). It is noteworthy that cultivars exhibited higher levels of polymorphism with a percent polymorphic loci (PPL) of 51.3% than landraces (PPL = 45.6%) and wild populations (PPL = 41.3%) (Supplementary Figure S1).

Country of origin-based pair-wise genetic differentiation analysis of allele frequency data was also carried out for the top five countries in the number of accessions (China, Germany, India, Italy, and Sweden). There was significant differentiation between all pairs of countries (*p* < 0.05). The highest and lowest differentiations were between India and Sweden (FST = 0.558) and between Germany and Sweden (FST = 0.133), respectively (Table 5). Pair-wise genetic differentiation analysis between accessions from different countries of origin based on the genotypic data for 22 SNP loci showed that accessions from Bangladesh were significantly differentiated from accessions from Canada, Finland, Germany, Italy, and Sweden (Table 6).

The genetic differentiation analysis carried out in terms of the accessions’ regions of origin (Africa, Asia, Europe, North America, and South America) based on allele frequency data revealed significant differentiation (*p* < 0.05) between all pairs of regions, except for the two South American accessions that were only significantly differentiated from Asian accessions. The highest differentiation was between African and European accessions (FST = 0.487) followed by differentiation between African and North American accessions (FST = 0.459). Although it was significant, differentiation between European and North American accessions was very low (FST = 0.072) (Table 5). The genetic differentiation analysis based on genotypic data of the 22 SNP loci between regions of germplasm origin was not significant (Table 6). 

The allele frequency-based analysis carried out in terms of the accessions’ germplasm providers (Jerrestad AB, CGN, IPK, NordGen, NPGS, and PGRC) revealed significant differentiation between all pairs except between accessions from Jerrestad AB and NordGen. The highest differentiation was between accessions from Jerrestad AB and CGN (FST = 0.538) followed by NordGen versus CGN (FST = 0.416) (Table 5). The significant differentiation of CGN accessions from Jerrestad AB and NordGen accessions was also evident when the genotypic data of 22 SNP loci was used. Additionally, genotypic data-based analysis revealed significant differentiation between accessions of CGN and IPK, NPGS and IPK, and NPGS and NordGen (Table 6).

### 3.4. Region and/or Country-Specific Alleles and the Effects on Their Corresponding Genes

Nine out of the five hundred and thirty-four SNP loci contained alleles that were unique to a particular region (region-specific alleles). All of them are minor alleles with an allele frequency ranging from 0.004 (at loci Bn-A01-p1001022 and Bn-A02-p20845905) to 0.176 (at locus NC_024795-2_25991653). Among nine alleles, four are intergenic, two are upstream, two are missense, and one is a downstream variant. The two missense variants (“A” allele at locus NC_024796-2_ 3347226 and “C” allele at locus NC_024800-2_ 3070370) were predicted to have moderate effects on their corresponding genes (A02p008020.1_BraROA and A06p008790.1_BraROA). Eight of the nine alleles were specific to Europe, while one was specific to North America (Table 7). Four of the nine alleles were specific to a single country within a region, while the remaining five were found in more than one country within a region (Table 7). Among the eight Europe-specific alleles, two were specific to France while one was specific to Italy. The North American-specific allele was unique to accessions from Cuba (Table 7).

## 4. Discussion

The 188 *B. rapa* accessions investigated in this study using 534 bi-allelic SNP markers include cultivars, landraces, breeding materials, and wild populations that represent Africa, Asia, Europe, Oceania, North America, and South America. SeqSNP-based genotyping was carried out utilizing a pool-seq technique described in Osterman et al. [45]. This study confirms the SeqSNP’s potential for target SNP genotyping as well as *de novo* SNP discovery (novel SNPs). However, it should be noted that the novel SNPs are located close to the target SNPs. Hence, the simultaneous use of target and novel SNPs is not ideal for QTL mapping and genome-wide association studies because of their genetic linkage. The markers used included both genic and non-genic markers distributed across all 10 chromosomes, with the number of markers per chromosome ranging from 39 (Chromosome A05) to 79 (Chromosome A03). The predicted effects of the markers on their associated genes varied from low to high.

### 4.1. Genic SNP Markers with Predicted Moderate to High Effects on Their Associated Genes

Among the SNP markers included in this present study, those found within coding sequences and those playing roles in gene splicing added up to 108. More than 60% of these markers had missense variants that were predicted to have moderate effects on their genes. These include two SNP loci (NC_024800-2_18977265 and Bn-A01-p2207452) within the *A06p035100.1_BraROA* and *A01p005290.1_BraROA* genes, respectively, which are homologs of Arabidopsis genes coding for pentatricopeptide repeat-containing (PPR) proteins At5g61800 and At4g33170, respectively. Proteins in this family are involved in various functions that include regulation of RNA metabolism, such as RNA splicing, editing, translation, and electron transport, as well as plant growth and response to biotic and abiotic stresses [46,47,48]. For example, mutant Arabidopsis plants with the reduced expression of a PPR gene, PPR40, were shown to be more sensitive to oxidative stress [46]. Although the minor allele frequency was very low at both loci, the minor allele at NC_024800-2_18977265 was distributed across Europe and Asia, while that of Bn-A01-p2207452 was limited to France. To determine the potential effects of mutation at these loci, it is worthwhile to examine accessions harboring each allele and compare them in terms of abiotic stresses, such as drought.

Another locus with missense variants was Bn-A10-p17148313, located on Chromosome 10 within *A10p035170.1_BraROA*, a gene that codes for a cytosolic pyruvate kinase. Pyruvate kinase catalyzes phosphoenolpyruvate (PEP) in the presence of ADP to pyruvate while producing ATP. In lipid biosynthesis, pyruvate is utilized as a source of ATP and acetyl-CoA. Both cytosolic (PKp) and plastidic (PKc) isoforms of pyruvate kinase exist. The activity and concentration of PKp correlate with the most active stage of lipid biosynthesis, while pyruvate generated by PKc can be imported and used directly for lipid biosynthesis [49,50]. Both alleles at the Bn-A10-p17148313 locus are widely distributed across continents. However, a significant number of accessions are fixed for each allele. Hence, it is interesting to investigate accessions containing each allele and compare them in terms of, for example, oil contents to determine the potential effects of mutation at this locus.

Mutations that result in splice_acceptor_variants that were predicted to have high effects on their genes were detected at two SNP loci (Bn-A03-p10732934 and NC_024800-2_3070398) within the *A03p027010.1_BraROA* and *A06p008790.1_BraROA* genes on chromosomes A03 and A06, respectively. These genes code for ribosomal RNA small subunit methyltransferase and Phosphatidylinositol 4-phosphate 5-kinase 7 (PIP5K7), respectively. Ribosomal RNA methylation plays a crucial role in the assembly and activity of ribosomes, which in turn play crucial roles in plant growth, development, and stress responses [51,52]. According to Ngoc et al. [52], mutant plants of the methyltransferase gene (*At3g28460*) had shorter roots, lower fresh weight, and pale green leaves, suggesting that this gene plays a role in adaptation to cold. 

A set of kinases and phosphatases regulate the synthesis and turnover of phosphoinositide (PI) molecules that are key regulators of a wide range of cellular processes, including signal transduction and cell proliferation [53,54,55]. For example, Wada et al. [54] showed that Phosphatidylinositol 4-phosphate 5-kinase (PIP5K) genes responded to PI deficiency by transducing signals to pathways involved in root growth. Kuroda et al. [55] reported that PIP5K7 is one of the PIP5K genes involved in root growth adaptation to hyperosmotic stress conditions. 

Among mutations predicted to have high effects on their genes were splice_donor_variants detected at three SNP loci (Bn-A03-p14474478, Bn-A06-p15059166, and Bn-A07-p19066968) within the *A03p036830.1_BraROA*, *A06p036240.1_BraROA*, and *A07p046640.1_BraROA* genes on chromosomes A03, A06, and A07, respectively. The first two genes code for uncharacterized proteins, while *A07p046640.1_BraROA* codes for protein detoxification 17 (DTX17). Detoxification proteins are a class of proteins that degrade both endogenous and exogenous toxins as well as reactive oxygen species (ROS). Detoxification proteins are produced by a large gene family [56], and most of them are produced in response to toxins’ stimulation [57]. Li et al. [56] demonstrated that the *Arabidopsis thaliana* protein detoxification 1 (AtDTX1) (for *Arabidopsis*) serves as an efflux carrier for plant-derived toxic compounds as well as detoxification of a heavy metal, cadmium. Since AtDTX17 was closely clustered with AtDTX1 [56] in the phylogenetic tree of genes in this family, AtDTX17 may also play a role in heavy metal detoxification. 

The Bn-A04-p15655213 locus, located on the chromosome *A04p030970.1_BraROA* gene, was found to contain a stop-lost mutation predicted to have a significant effect on the gene. The gene codes for a homolog of Arabidopsis’ putative F-box protein At4g22660, which contains the DUF295 domain. According to Gong et al. [58], Abscisic acid (ABA) and abiotic stresses suppress F-box/DUF295 expression in Arabidopsis. This gene plays a role in the ABA-mediated inhibition of seed germination and seedling growth. 

Interestingly, both alleles at each of the two loci with splice_acceptor_variants, three loci with splice_donor_variants, and one locus with stop_lost_variants were widely distributed across accessions from different geographical regions despite the fact that a significant number of accessions were fixed for either of the two alleles at each locus. Hence, it would be worthwhile to group accessions according to their alleles at these loci and compare their performance with regard to the traits their corresponding genes regulate.

### 4.2. SNP Loci with Region/Country Specific Alleles 

The accessions containing country/region-specific alleles are highly valuable genetic resources and should be included in gene bank core collections [59,60], and the sites of their original collection should be prioritized for in situ conservation. Among the nine SNP loci carrying alleles specific to a particular region/country, two (NC_024796-2_ 3347226 and NC_024800-2_ 3070370) had missense variants within their corresponding genes (*A02p008020.1_ BraROA* and *A06p008790.1_ BraROA*, respectively). However, only *A06p008790.1_BraROA* codes for a protein with known functions. It codes for phosphatidylinositol 4-phosphate 5-kinase 7. Two SNP loci (Bn-A01-p1001022 and NC_024803-2_35208073) were located at the upstream regions of genes that encode for known proteins. These loci are located upstream of *A01p002320.1_BraROA* and *A09g511940.1_BraROA*, which code for the homeobox-leucine zipper protein ATHB-40 and UDP-glycosyltransferase 76F1, respectively.

The missense variant (allele) within *A06p008790.1_BraROA* that codes for phosphatidylinositol 4-phosphate 5-kinase 7 with a predicted moderate effect on the gene was specific to Italy, as it was observed only in four of the twenty-eight Italian accessions. Two of these accessions (PI633168 and PI662794) were obtained from NPGS (USA), while the other two (CR2289 and CR2203) were obtained from IPK (Germany). Interestingly, PI633168 is a cultivar, CR2203 is a landrace, and CR2289 is a wild population. This allele was found in 14.3% of Italian accessions included in this present study. Given that the allele is found in different Italian population types, it is likely that the original source of the allele is an Italian wild population. Hence, it is worth checking the pedigree of cultivar PI633168. Phosphatidylinositol 4-phosphate 5-kinase 7 is involved in plant adaptation to abiotic stresses, such as phosphorous deficiency [54,55], and hence, it is interesting to compare these accessions with other accessions lacking the mutant allele under abiotic stress conditions. A SNP locus located upstream of the *homeobox-leucine zipper protein ATHB-40* gene had a rare allele specific to an accession from France provided by IPK (CR2468). The ATHB-40 transcription factor is a member of the HD-Zip family involved in plant growth hormone regulation [61,62]. Studies have shown that ATHB-40 is among genes upregulated under stress conditions, such as drought and salinity [63,64].

The NC_024803-2_35208073 locus located upstream of the UDP-glycosyltransferase 76F1 gene had an allele specific to Europe, as the allele was only found in four and three accessions from Germany and Italy, respectively. This allele had a very low frequency (0.03). UDP-glycosyltransferase is one of the key enzymes of glycosylation and plays crucial roles in stress responses, such as plant resistance to pathogen infection [65] as well as in plant growth [66]. Hence, specific genotypes homozygous for the rare allele in these accessions need to be used in crossbreeding to evaluate its possible phenotypic effect through association with alleles in the *UDP-glycosyltransferase 76F1* gene or the expression levels of this gene.

*A01p046190.1_BraROA*, which codes for the pollen receptor-like kinase 4 (PRK4), is the closest gene to the SNP locus (NC_024795-2_25991653) containing an allele specific to Cuban accessions. This allele was found in all three accessions from Cuba. Accessions CR2231 and CR3452 are landraces provided by IPK (Germany), while PI633169 is a cultivar provided by NPGS (USA). This suggests that the allele may be widely distributed in Cuban turnip rape genetic resources. The locus is located 6.4 kb downstream of the *A01p046190.1_BraROA* gene (A01: 25998064-26002028; reverse strand) and could be in linkage disequilibrium (LD) with this gene. PRK4 is involved in pollen tube function through mediating extracellular signals that organize cytoskeleton orientation during polar pollen tube growth [67,68]. A comparison of these Cuban accessions with others in terms of pollen tube growth and fertilization may provide insight into the potential effect of the mutant allele. 

### 4.3. Genetic Relationship between Accessions 

The genetic relationship between the 188 accessions that represent global collections of turnip rape was evaluated through cluster and principal coordinate analyses. In cluster analysis, the majority of the accessions (81.9%) were grouped into three major clusters, while 18.1% were found to be solitary or formed minor clusters. The 18 accessions in cluster-I are the most distantly related accessions to all other accessions, and 72.2% of them are of Asian origin. The 34 solitary and minor-cluster accessions were also distantly related to those in cluster-II and cluster-III, and over two-thirds of them were of Asian origin. Interestingly, only four Asian accessions were clustered in cluster-II. Cluster-II and cluster-III, combined, accounted for 72.3% of the studied accessions, totaling 136 accessions. Accessions in cluster-II and cluster-III are more closely related to each other than to any other accessions. It is noteworthy that all accessions from North and South America and 88.5% of accessions from Europe were grouped in cluster-II or cluster-III.

The result of principal coordinate analysis (PCoA), which explained about two-thirds of the total variation among accessions, is in general agreement with that of cluster analysis. PCoA grouped the accessions into three clusters along the *x*-axis (PCo1), which explained over half of the total variation, and the clustering pattern corresponded well to those obtained via cluster analysis. The major difference was that accessions in cluster-I of the NJ tree were separated into cluster-I and cluster-II in PCoA. Also, the PCoA analysis did not resolve accessions in cluster-I and cluster-II as well as those in the minor clusters or that were solitary since they all clustered in the PCoA cluster-III, except for seven accessions. This is expected, as the PCoA bi-plot explained only two-thirds of the total variation.

Overall, the results demonstrated that Asian turnip rape accessions are more distantly related to accessions from other geographical regions, which are more closely related to each other than to Asian accessions. Based on the distribution of accessions in the NJ tree and PCoA bi-plot, it appears that Asia has the highest level of genetic diversity for turnip rape, followed by Europe. However, the lesser genetic diversity of North American turnip rape compared to Asia and Europe may be attributable to the fact that there were fewer accessions from North America than from Asia and Europe.

As indicated in the materials and methods section, 94 of the 188 accessions used in this present study were cultivars. Of the 94 cultivars included in this present study, 67 (71.3%) were clustered in cluster-III of the NJ tree (Figure 3), which accounted for 90.5% of the accessions in this cluster. Furthermore, all cultivars obtained from NordGen were clustered in cluster-III except for one accession. The results suggest that most cultivars, particularly those from Europe and North America, are closely related even though they were obtained from different germplasm providers. The fact that European and American cultivars are more similar to each other than their similarities to Asian cultivars indicates that American cultivars are descendants of *B. rapa* domesticated in Europe, as suggested by McAlvay et al. [5]. Furthermore, it may indicate an exchange of cultivars between countries; for example, within Europe as well as between Europe and the USA. Such high genetic similarity between the cultivars could also be partly because they were bred for similar traits across the world. In this regard, the high genetic similarity between the 13 Jerrestad AB cultivars suggests they may be similarly vulnerable to biotic and abiotic stresses. Therefore, crossbreeding with genetically distant elite cultivars may enhance the genetic diversity of the breeding program’s germplasm, thereby providing the opportunity for breeding for resistance/tolerance to these stresses.

Despite the high level of overall similarity between cultivars, 28.7% of them (27 accessions) were widely distributed across branches outside cluster-I. Among 71 cultivars of European origin, seven were the most distinct, all obtained from IPK. Six of these cultivars belong to Germany, while one belongs to Sweden. Provided that they possess desirable characteristics for major traits, such as seed yield and oil content, they can be prioritized for crossbreeding with cultivars in cluster-I as a means to develop new cultivars superior to existing ones in terms of these desirable traits. None of the 12 Asian cultivars were placed in cluster-I, suggesting their distinctness from most European and North American cultivars. Previous studies have also demonstrated a clear differentiation between Asian and European turnip rape [4,69]. Therefore, crossbreeding between Asian and European cultivars may be an effective approach since genetic recombination obtained from such crossbreeding may result in superior cultivars. 

Among the landrace accessions, 80.5% were clustered in cluster-II, suggesting high genetic similarity between most landrace accessions, similar to the case in cultivars. With a few exceptions, European cultivars are genetically more similar to European landraces than to Asian cultivars. Similarly, Asian cultivars are more genetically similar to Asian landraces than to European cultivars. These suggest that European and Asian cultivars were predominantly derived from European and Asian landraces, respectively. However, such a suggestion cannot be made for European versus North American cultivars and landraces. This is probably because both European and American cultivars were developed from landraces belonging to the same gene pool, in line with previous research [4,5]. Another interesting observation was that most wild populations were placed in cluster-II, a cluster in which over 50% of landrace accessions were placed. This suggests that most landrace accessions are genetically more similar to wild populations than to advanced cultivars. Hence, landraces could serve as a potential source of desirable alleles for elite cultivar improvement.

### 4.4. Analysis of Molecular Variance and Genetic Differentiation between Various Groups

As revealed by the analysis of molecular variance (AMOVA) conducted based on allele frequency and genotypic data, there exist highly significant genetic differences between the turnip rape genetic resources of different countries and continents as well as between accessions kept in different gene banks. However, genetic differentiation was higher among countries and continents than among gene banks. Such significant genetic differentiation between turnip rape cultivars from different regions was previously reported [69]. The lower differentiation between gene banks could be partly because some of the accessions obtained from different gene banks may belong to the same germplasm (are duplicates), as germplasm duplications could occur both within and across gene banks [70,71,72,73]. This can be exemplified by the two Canadian cultivars CR1621 and ACSC29 obtained from IPK (Germany) and A&A Canada, respectively, which were placed together at the end of cluster-III of the NJ tree (Figure 3). The fact that gene banks maintain overlapping germplasm from multiple countries is another possible explanation. As a result, a lower degree of genetic differentiation between germplasm from different gene banks than between germplasm from different countries of origin is expected to be observed. 

Allele frequency-based pair-wise genetic differentiation analysis between turnip rape accessions from five countries representing two continents (Europe and Asia) revealed significant differentiation among all pairs of countries. However, the levels of differentiation differed and showed interesting patterns. It appeared that the levels of differentiation between German accessions and accessions from the other four countries (China, India, Italy, and Sweden) were lower than the levels between accessions from these four countries. The results may suggest that turnip rape germplasm of German origin has been more commonly used for breeding in both European and Asian countries than turnip rape germplasm from other countries of origin. However, this should be confirmed through further research. A higher level of genetic differentiation was also observed between Indian and European turnip rape germplasm than between Chinese and European germplasms, suggesting greater gene flow between the latter pair than between the former. This is expected, as there has been a long history of germplasm exchange between continents, which continues to this day through export for industrial purposes, research, and preservation [74]. Overall, there was a higher degree of genetic differentiation between European and Asian turnip rape genetic resources than between genetic resources within each continent, in agreement with previous studies on different *B. rapa* morphotypes [4,26,69,75]. Differences in the number and types of accessions appear to have significant effects on genetic differentiation between accessions from different continents. As a result, only comparisons between Asia, Europe, and North America with comparable data sets were considered suitable, as they were more or less consistent across different analyses. Low genetic differentiation exists between European and North American germplasm, which have similar levels of differentiation from Asian turnip rape germplasm. 

Significant genetic differentiation exists between germplasm held by different gene banks. Among them, CGN (The Netherlands) has the most distinct turnip rape collection, while NPGS (USA) and IPK (Germany) have the least distinct collections on average. The absence of significant genetic differentiation between the Jerrestad AB cultivars (Sweden) and the collections in NordGen indicates that the Jerrestad AB turnip rape breeding program is based on turnip rape genetic resources of the Nordic region. Hence, it would be beneficial for the breeding company to incorporate germplasm from other gene banks, such as CGN, to broaden the gene pool for breeding.

Genetic differentiation between cultivars, landraces, and wild populations was also highly significant, as it accounted for about 17% of the total genetic variation. Pair-wise differentiation analysis revealed that cultivars were significantly differentiated from landraces and cultivars. Interestingly, no significant differentiation between landraces and wild populations was observed. This could be because of a high rate of gene flow between wild populations and landraces [76] since turnip rape is an outcrossing crop, and measures to minimize gene flow between wild populations and landraces are minimal, unlike the case for cultivars. It is also interesting to note that higher genetic variation exists within cultivars than within landraces and wild populations, as the percent polymorphic loci (PPL) of the cultivars was higher than that of the landraces and wild populations, suggesting that breeding did not lead to loss of genetic diversity in turnip rape. Previous research also showed that breeding for the improvement of the nutritional quality of European and Chinese *B*. *rapa* cultivars did not result in a significant reduction in genetic diversity [69,77]. Wild populations had the lowest PPL than both cultivars and landraces, indicating that they have the lowest genetic variation within populations on average. This suggests that domestication and breeding facilitate the introgression of novel alleles from various sources into populations targeted by humans for improvement.

## 5. Conclusions

The SNP markers used to estimate the genetic relationship between various groups of turnip rape accessions representing its global collection revealed highly informative variation patterns. The predicted effects of the genic markers on their associated genes varied from low to high. A number of missense and splice-acceptor/donor/stop-lost variants were predicted to have moderate and high effects on their genes, respectively. To determine whether these alleles exert any observable effects, it would be useful to group accessions based on their alleles at these loci and compare their phenotypic variation. Accessions containing alleles specific to a region or country identified in this study are highly valuable genetic resources that should be included in gene bank core collections. Additionally, comparative phenotypic characterization could identify desirable traits in these accessions. European and North American cultivars have higher genetic similarities to each other than to Asian cultivars. Hence, turnip rape improvement could be achieved by crossbreeding Asian and European cultivars through recombination of desirable alleles. Differentiation between cultivars and landraces was significant but not between landraces and wild populations. Since landraces are genetically closer to wild populations than to advanced cultivars, they may provide desirable alleles relevant to cultivar improvement. Interestingly, breeding did not lead to genetic erosion globally, as cultivars showed more genetic variation than landraces or wild populations. There is high genetic differentiation among turnip rape genetic resources from different countries and continents as well as gene banks. However, the differentiation was higher between countries and continents than between gene banks. CGN has the most distinct turnip rape collection, while NPGS and IPK have the least. The Jerrestad AB cultivars and the NordGen collections showed no significant genetic differentiation, indicating that Swedish turnip rape breeding relies on Nordic genetic resources. In addition, Swedish turnip rape cultivars have high genetic similarity. Hence, crossbreeding with genetically distant cultivars could enhance the gene pool’s diversity and facilitate the development of superior cultivars with desired traits.

## Figures and Tables

**Table 1:** Number of turnip rape accessions, representing different continents obtained from different gene banks and breeding companies, used in this study.

GenBank	Africa	Asia	Europe	North America	Oceania	South America	Unknown	Total
NordGen	0	0	33	1	0	0	0	34
Jerrestad AB	0	0	13	0	0	0	0	13
PGRC	0	24	3	3	0	0	0	30
CGN	0	7	1	2	0	0	1	11
IPK	1	8	64	8	1	2	2	86
NPGS	1	2	8	1	0	0	0	12
A&A Canada	0	0	0	2	0	0	0	2
Total	2	41	122	17	1	2	3	188

**Table 2:** The number and effects of SNPs within genes across the ten spring turnip rape chromosomes.

Consequence_Effect	Chromosome										Total
	A01	A02	A03	A04	A05	A06	A07	A08	A09	A10	
Missense variant_Moderate	12	5	9	5	0	9	7	6	0	13	66
Synonymous variant_Low	4	0	1	4	1	0	4	2	0	5	21
Splice polypyrimidine tract variant_Low	0	1	1	0	0	0	0	0	1	1	4
Splice donor variant_High	0	0	1	0	0	1	1	0	0	0	3
Splice polypyrimidine tract variant/Intron variant_Low	0	0	0	1	0	1	0	0	1	0	3
Splice acceptor variant_High	0	0	1	0	0	1	0	0	0	0	2
Splice donor fifth base variant/Intron variant_Low	0	0	0	1	0	0	0	0	0	1	2
Splice donor region variant/Intron variant_Low	0	0	1	0	0	1	0	0	0	0	2
Splice region variant/Synonymous variant_Low	0	0	1	0	0	1	0	0	0	0	2
Missense variant/stop retained variant_Moderate	0	0	1	0	0	0	0	0	0	0	1
Splice region variant/Intron variant_Low	0	0	0	0	0	0	1	0	0	0	1
Stop lost_High	0	0	0	1	0	0	0	0	0	0	1
Total	16	6	16	12	1	14	13	8	2	20	108

Note: only intron variants that have roles in gene splicing were included here.

**Table 3:** Analysis of molecular variance (AMOVA) based on allele frequencies of SNPs at 534 bi-allelic loci for different groups of turnip rape accessions grouped according to their germplasm providers, population types, countries of origin, or regions (continents) of origin.

Source of Variation	Degrees of Freedom	Sum of Squares	Variance Components	Percentage of Variation	Fixation Index	*p*-Value
Among accession groups ^a^	5	2.311	0.015	19.0	Phi_PT_ = 0.194	0.001
Within accession groups ^a^	180	11.230	0.062	81.0		
Total	185	13.542	0.077			
Among accession groups ^b^	2	0.954	0.012	17.5	Phi_PT_ = 0.175	0.001
Within accession groups ^b^	139	8.108	0.058	82.5		
Total	141	9.063	0.071			
Among accession groups ^c^	4	2.227	0.025	38.0	Phi_PT_ = 0.378	0.001
Within accession groups ^c^	112	4.590	0.041	62.0		
Total	116	6.817	0.066			
Among accession groups ^d^	4	2.332	0.023	27.0	Phi_PT_ = 0.268	0.005
Within accession groups ^d^	179	11.025	0.062	73.0		
Total	183	13.357	0.084			

^a^ = 186 accessions were grouped into six groups based on their germplasm provider; ^b^ = 142 accessions were grouped into three groups based on their population type; ^c^ = 117 accessions were grouped into five groups based on their country of origin; ^d^ = 184 accessions were grouped into five groups based on their region (continent) of origin. Note: allele frequencies at 534 bi-allelic SNP loci were used to calculate Nei’s unbiased genetic distance, which was then used for the analysis of molecular variance. One thousand permutations were used during the analysis.

**Table 4:** Analysis of molecular variance (AMOVA) based on the genotypic data of 22 bi-allelic SNP loci that lack heterozygosity for different numbers of turnip rape accessions grouped according to their germplasm providers, population types, countries of origin, or regions (continents) of origin.

Source of Variation	Degrees of Freedom	Sum of Squares	Variance Components	Percentage of Variation	Fixation Index	*p*-Value
Among accession groups ^a^	6	58.4	0.38 Va	23.49	F_ST_ = 0.24	0.003
Within accession groups ^a^	175	217.0	1.24 Vb	76.51		
Total	181	275.4	1.62			
Among accession groups ^b^	2	9.0	0.15 Va	17.08	F_ST_ = 0.17	0.03
Within accession groups ^b^	145	105.6	0.73 Vb	82.92		
Total	147	114.7	0.88			
Among accession groups ^c^	7	100.3	0.85 Va	51.07	F_ST_ = 0.51	0
Within accession groups ^c^	150	122.8	0.82 Vb	48.93		
Total	157	223.1	1.67			
Among accession groups ^d^	3	96.3	1.47 Va	58.97	F_ST_ = 0.59	0
Within accession groups ^d^	170	174.1	1.02 Vb	41.03		
Total	173	270.4	2.49			

^a^ = Ninety-one accessions were grouped into seven groups based on their germplasm provider; ^b^ = seventy-four accessions were grouped into three groups based on their population type; ^c^ = seventy-nine accessions were grouped into eight groups based on their country of origin; ^d^ = eighty-seven accessions were grouped into four groups based on their region (continent) of origin. Note: one thousand permutations were used during the analysis.

**Table 5:** Pair-wise differentiation between different groups of turnip rape accessions grouped according to their germplasm provider, population type, country of origin, or region (continent) of origin based on allele frequencies of SNPs at 534 bi-allelic loci.

Accession-group ^a^	Cultivar	Landrace	Wild			
Cultivar	-					
Landrace	0.188 *	-				
Wild	0.208 *	0.040	-			
Accession-group ^b^	China	Germany	India	Italy	Sweden	
China	-					
Germany	0.188 *	-				
India	0.243 *	0.368 *	-			
Italy	0.340 *	0.234 *	0.475 *	-		
Sweden	0.466 *	0.133 *	0.558 *	0.388 *	-	
Accession-group ^c^	Africa	Asia	Europe	North America	South America	
Africa	-					
Asia	0.276 *	-				
Europe	0.487 *	0.308 *	-			
North America	0.459 *	0.334 *	0.072 *	-		
South America	0.198	0.317 *	0.174	0.146	-	
Accession-group ^d^	Jerrestad AB	CGN	IPK	NordGen	NPGS	PGRC
Jerrestad AB	-					
CGN	0.538 *	-				
IPK	0.169 *	0.363 *	-			
NordGen	0.088	0.416 *	0.105 *	-		
NPGS	0.281 *	0.194 *	0.072 *	0.187 *	-	
PGRC	0.407 *	0.172 *	0.167 *	0.243 *	0.076 *	-

^a^ = 142 accessions were grouped into three groups based on their population type; ^b^ = 117 accessions were grouped into five groups based on their country of origin; ^c^ = 184 accessions were grouped into five groups based on their region (continent) of origin; ^d^ = 186 accessions were grouped into six groups based on their germplasm provider; * = significant differentiation between the groups (*p* < 0.05). Note: allele frequencies at 534 bi-allelic SNP loci were used to calculate Nei’s unbiased genetic distance, which was then used for differentiation analysis between groups of accessions. One thousand permutations were used during the analysis.

**Table 6:** Pair-wise FST between different groups of accessions for different numbers of turnip rape accessions grouped according to their germplasm provider, population type, country of origin, or region (continent) of origin based on the genotypic data of 22 bi-allelic SNP loci that lack heterozygosity.

Accession Group ^a^	Cultivar	Landrace	Wild					
Cultivar	-							
Landrace	0.215 *	-						
Wild	0.109	0.121	-					
Accession group ^b^	Africa	Asia	Europe	North America				
Africa	-							
Asia	0.599	-						
Europe	0.521	0.835	-					
North America	0.674	0.873	−0.007	-				
Accession group ^c^	Jerrestad AB	A&A Canada	CGN	IPK	NordGen	NPGS	PGRC	
Jerrestad AB	-							
A&A Canada	−0.151	-						
CGN	0.495 *	0.271	-					
IPK	−0.021	−0.097	0.635 *	-				
NordGen	0.010	−0.077	0.461 *	0.021	-			
NPGS	0.441	0.234	0.221	0.500 *	0.268 *	-		
PGRC	0.041	−0.113	0.310	0.088	0.020	0.153	-	
Accession group ^d^	Austria	Bangladesh	Canada	Finland	Germany	Italy	Sweden	USA
Austria	-							
Bangladesh	0.953	-						
Canada	−0.125	0.950 *	-					
Finland	−0.045	0.792 *	0.038	-				
Germany	0.261	0.987 *	0.196	0.028	-			
Italy	0.096	0.931 *	0.157	0.133	0.480	-		
Sweden	−0.089	0.908 *	−0.027	0.036	0.054	0.143	-	
USA	0.040	0.951	0.106	0.049	0.650	−0.078	0.078	-

^a^ = Seventy-four accessions were grouped into three groups based on their population type; ^b^ = eighty-seven accessions were grouped into four groups based on their region (continent) of origin; ^c^ = ninety-one accessions were grouped into seven groups based on their germplasm provider; ^d^ = seventy-nine accessions were grouped into eight groups based on their country of origin; * = significant differentiation between the groups (*p* < 0.05).

**Table 7:** List and description of SNP loci with region and/or country-specific alleles (private alleles), including allele effects and corresponding genes and proteins.

	Region Specific Allele								
	Europe/France	Europe/France	Europe/ **	Europe/Italy	Europe/ **	Europe/ **	Europe/ **	Europe/ **	North America/Cuba
Locus	Bn-A01-p1001022	Bn-A02-p20845905	NC_024796-2_ 3347226	NC_024800-2_ 3070370	NC_024803-2_ 35208073	NC_024804-2_15703515	NC_024803-2_33076115	NC_024803-2_30767803	NC_024795-2_25991653
Chromosome	A01	A02	A02	A06	A09	A10	A09	A09	A01
SNP position	1061243	24397768	3347226	3070370	35208073	15703515	33076115	30767803	25991653
Ref/Alt alleles	A/C	A/C	T/A	T/C	C/T	C/T	T/C	G/T	C/T
PA	C	C	A	C	T	T	C	T	T
PA freq	0.004	0.004	0.021	0.017	0.030	0.017	0.025	0.012	0.176
PA description	Upstream gene variant	Intergenic variant	Missense variant	Missense variant	Upstream gene variant	Downstream gene variant	Intergenic variant	Intergenicvariant	Intergenic variant
PA effect	Modifier	Modifier	Moderate	Moderate	Modifier	Modifier	Modifier	Modifier	Modifier
PA-associated gene	A01p002320.1_ BraROA	A02g509130.1_ BraROA *	A02p008020.1_ BraROA	A06p008790.1_ BraROA	A09g511940.1_ BraROA	A10p024240.1_ BraROA	A09g510750.1_BraROA *	A09g510320.1_BraROA *	A01p046190.1_BraROA *
PA-associated Protein	homeobox-leucine zipper protein ATHB-40	-	uncharacterized	phosphatidylinositol 4-phosphate 5-kinase 7	UDP-glycosyltransferase 76F1	uncharacterized	uncharacterized	uncharacterized	Pollen receptor-like kinase 4
Coding strand	Forward	Forward	Forward	Reverse	Forward	Reverse	Forward	Reverse	Reverse
Codon change	-	-	Ttc/Atc	gAg/gGg	-	-	-	-	-
AA change	-	-	Phe/Ile	Glu/Gly	-	-	-	-	-

* = the closest gene; ** = the allele was found in more than one country within the region; AA change = amino acid change; Phe/Ile = phenylalanine/isoleucine; Glu/Gly = glutamic acid/glycine.

## Data Availability

The analyzed data are presented in the article and the Supplementary Material. Any further inquiries can be directed to the corresponding author.

## Supplementary Materials

The following supporting information can be downloaded at: https://www.mdpi.com/article/10.3390/genes15091187/s1, Figure S1: Bar graphs showing the number of known cultivars, landraces, and wild populations used in this present study and their corresponding mean percent polymorphic loci; Table S1: List of 108 SNPs found within 72 *Brassica rapa* genes with various descriptions, including their positions within genes and coding sequences, mutation types, and predicted effects of those mutations.
