# Supplementary figures and images for: SeqSNP-Based Targeted GBS Provides Insight into the Genetic Relationships among Global Collections of Brassica rapa ssp. oleifera (Turnip Rape)
Source: Genes (Basel). 2024 Sep 10;15(9):1187. doi: 10.3390/genes15091187 (PMC11431370; doi:10.3390/genes15091187)

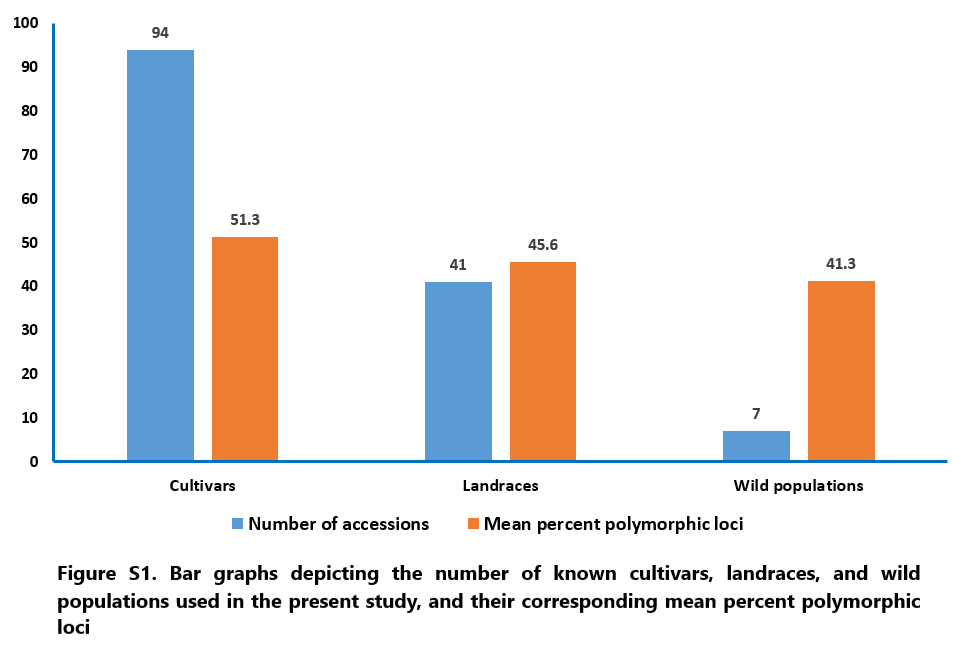

Supplement: Supplementary file 1 [file genes-15-01187-s001.zip › Figure S1.tiff]
